# The Rapid Initiation, Titration, and Transition from Intravenous to Oral Treprostinil in a Patient with Severe Pulmonary Arterial Hypertension

**DOI:** 10.1155/2015/498981

**Published:** 2015-09-17

**Authors:** James Benjamin Gleason, Justin Dolan, Pirouz Piran, Franck Farzad Rahaghi

**Affiliations:** ^1^Department of Pulmonary and Critical Care Medicine, Cleveland Clinic Florida, 2950 Cleveland Clinic Boulevard, Weston, FL 33331, USA; ^2^Department of Internal Medicine, Cleveland Clinic Florida, 2950 Cleveland Clinic Boulevard, Weston, FL 33331, USA

## Abstract

In patients who require urgent initiation of pulmonary arterial hypertension medications due to disease progression, it is customary to start intravenous prostacyclin therapy, typically during a hospital admission. If there are complicating factors or relative contraindications to intravenous and subcutaneous prostanoids, oral treprostinil provides another pathway to prostanoid therapy, but this usually requires a prolonged titration. We describe the case of a thirty-six-year-old male with severe pulmonary arterial hypertension and contraindication to intravenous and subcutaneous prostanoid therapy due to congenital deafness and the risk of not hearing the intravenous pump alarms. Intravenous treprostinil was initiated, titrated to high dose, and then rapidly transitioned to oral treprostinil. A rapid initiation, titration, and transition from intravenous to oral treprostinil can be safely performed under watchful supervision in order to achieve higher and more efficacious doses of oral treprostinil in a timely manner.

## 1. Introduction

Treprostinil is a tricyclic benzidine prostanoid approved for the treatment of World Health Organization (WHO) Group 1, Functional Classes II–IV pulmonary arterial hypertension (PAH). There are multiple routes of administration including continuous intravenous (IV) infusion, continuous subcutaneous infusion, inhalation, and most recently oral sustained-release osmotic tablet. In advanced pulmonary hypertension, it is reasonable to admit the patient to the hospital and start IV or subcutaneous prostanoid therapy. In patients with relative contraindication or complicating factors precluding intravenous prostanoids, oral treprostinil is an option, but titration typically involves a prolonged duration. Currently, guidelines do not exist for the rapid initiation and titration of IV treprostinil with early rapid transition to oral treprostinil. We describe the case of a thirty-six-year-old male with severe pulmonary arterial hypertension who was successfully started on IV treprostinil, titrated to high dose, and then rapidly transitioned to oral treprostinil on a background of ambrisentan and sildenafil. This was performed with close inpatient monitoring.

## 2. Case Presentation

The patient is a thirty-six-year-old male with a history of congenital rubella with sensorineural deafness, partial blindness, patent ductus arteriosus complicated by Eisenmenger's syndrome, and the development of PAH prior to surgical repair at the age of two. Additionally, he also suffers from right sided heart failure, atrial fibrillation, restrictive ventilatory defect with hypoventilation, and obstructive sleep apnea requiring nightly noninvasive positive pressure ventilation. He was originally diagnosed with WHO Group I Class II PAH in 2008, and at that time he began treatment with sildenafil and ambrisentan. The disease progressed and he was initiated on inhaled treprostinil. Due to recent worsening of symptoms, more consistent with WHO Classes III-IV, he was admitted to the hospital for further workup. Echocardiogram revealed evidence of worsening pressures, severe diastolic right heart failure, moderate right ventricular dilation, systolic right ventricular dysfunction, and a large pericardial effusion with pretamponade physiology presumably related to his PAH and concurrent warfarin use. The pericardial effusion was managed by pericardiocentesis and pigtail catheter placement draining more than 1 liter of bloody fluid. We felt that the severity of his PAH needed to be reevaluated and that he may benefit from more aggressive therapy with a prostacyclin. Right heart catheterization (RHC) was repeated and he was found to have a mean pulmonary artery pressure (mPAP) of 47 mm Hg. Due to poor wedge waveforms, he underwent left heart catheterization (LHC) demonstrating the left ventricular end diastolic pressure (LVEDP) to be 14 mm Hg and cardiac output (CO) to be 5.7 L/min. Further calculations exposed a transpulmonary pressure gradient (TPG) of 33 mm Hg and pulmonary vascular resistance (PVR) of 5.7 mmHg*∗*min/L (Woods units) despite treatment with sildenafil, ambrisentan, and inhaled treprostinil.

**Figure 1:**
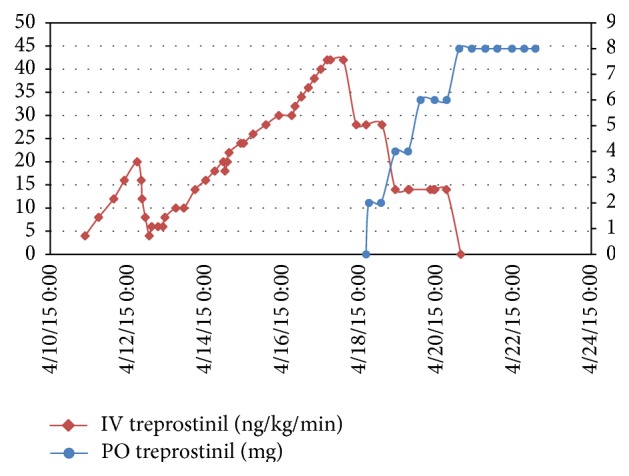
Graphical representation of the rapid titration of intravenous treprostinil (red line) over seven days followed by a stepwise dose reduction while transitioning to oral treprostinil (blue line).

He was started on IV treprostinil (Figure 1) with an initial dose of 4 ng/kg/min which was increased by 4 ng/kg/min every 8 hours. Within 36 hours, the dose was 20 ng/kg/min, but he developed hypotension requiring phenylephrine temporarily. We reduced the IV treprostinil but the etiology of this hypotension was believed to be infectious due to concurrent leukocytosis and fever. Fortunately, these issues resolved with broad spectrum antibiotics and cultures remained negative allowing treprostinil to be increased to 42 ng/kg/min over the next 96 hours. After demonstrating hemodynamic stability at this dose, we began the transition to oral treprostinil.

We reduced the IV treprostinil from 42 ng/kg/min to 28 ng/kg/min one hour after starting oral treprostinil 2 mg every 8 hours. After three doses of 2 mg, the oral treprostinil was increased to 4 mg and one hour later IV treprostinil was reduced to 14 ng/kg/min. Oral treprostinil was continued at 4 mg every 8 hours for three doses and then increased to 6 mg every 8 hours. After three doses of 6 mg, oral treprostinil was increased to 8 mg and one hour later IV treprostinil was discontinued. The patient tolerated the transition without any jaw pain, headache, flushing, nausea, or abdominal pain. He did have loose stools that were controlled with loperamide. After close monitoring for the subsequent twenty-four hours, he was discharged home with close outpatient follow-up.

## 3. Discussion

Several agents have been used to treat pulmonary arterial hypertension; these include endothelin receptor antagonists, phosphodiesterase type 5 inhibitors, stimulators of soluble guanylate cyclase, and prostacyclins. Of these different classes, prostacyclins have been most associated with improvement in functional capacity [1–4] and ultimately survival [5] and are recommended for treatment of WHO Functional Class IV patient. Chronic administration of IV prostacyclins requires an in-dwelling central venous catheter which increases the risk of acquiring a blood stream infection. Subcutaneous delivery does not require invasive catheter placement but still uses a pump and is associated with issues concerning the management of pain or infection at the infusion site [6]. In our particular case, these delivery methods were undesirable due to the visual and auditory impairments afflicting our patient which could prevent him from identifying problems with the infusion apparatus. Because of these limitations, we chose to initiate therapy with IV treprostinil while closely monitoring hemodynamic parameters followed by rapid transition to oral treprostinil.

Treprostinil diolamine is administered by an osmotic sustained release tablet dosed every 8 to 12 hours. This oral formulation has a bioavailability of 17% [7] and achieves peak serum concentration within 8 hours, increasing linearly with higher doses [8]. Metabolism occurs in the liver and for this reason it is contraindicated with liver failure; in patients with milder liver dysfunction, risks include increased total exposure [9]. The recommended initiation dose of oral treprostinil is 0.25 mg every twelve hours, though three times a day titration as we did is thought to be easier for patient to tolerate. Every three to four days the dose can be increased by 0.25 or 0.50 mg increments as the patient tolerates [7]. In safety trials of oral treprostinil monotherapy, the mean tolerated dose after twelve weeks was 3.4 mg twice per day [10] and the reported side effects included headache, diarrhea, nausea, flushing, and jaw pain. Currently, we believe that IV treprostinil is most efficacious at higher doses which correspond to oral doses of 6–10 mg every 8 hours. We believe that the probable 2–6 months it would have taken to achieve these equivalent doses was not acceptable for our patient [11, 12].

After successful transition and up-titration, our patient reported improved exercise capacity. His subjective reports of this early to medium term benefit were harmonious with the pivotal treprostinil trials which showed significant improvement in clinical symptom scores and six-minute walk tests correlating with higher doses [6]. Removal of the pulmonary artery catheter was hastened due to the development of transient sepsis type symptoms consequently limiting our hemodynamic data on higher doses of treprostinil. Nevertheless, these same trials only demonstrated minor changes in hemodynamics after 12 weeks [6].

Transitioning from one prostanoid delivery method to another may be necessary in scenarios where there are delivery related side effects such as cough and pain or complications like catheter related infections. In our patient, transition was expedited due to the potential hazards of outpatient administration associated with the sensory deficits related to his congenital rubella infection. The most studied transition has been from IV epoprostenol to parenteral or subcutaneous treprostinil; this usually occurs in the hospital by slowly increasing and decreasing each respective agent over the course of a few days [13–15]. When transitioning from IV treprostinil to oral treprostinil, there are no published consensus guidelines or suggested regimens to guide providers yet, though the results of the TDE-PH-205 trial, presented previously, suggest that this conversion can be done in approximately 5 days, using one mg of oral treprostinil every 8 hours for 10 ng/kg/min of intravenous or subcutaneous treprostinil [16, 17].

Our patient was already being treated with three-drug combination therapy including inhaled treprostinil prior to the initiation of IV therapy. A recent case series demonstrated that rapid titration of IV treprostinil in prostanoid naïve patients was safe [18]. We strongly believe that the method we describe can be used successfully for the prostanoid-naïve to equal or possibly even greater effect with close inpatient monitoring.

## 4. Conclusion

The rapid initiation and titration of IV treprostinil followed by rapid transition from IV to oral treprostinil may be safely performed under the guidance of experienced physicians with close inpatient monitoring.
